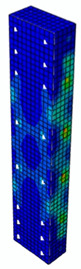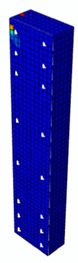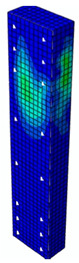# Nonlinear Analysis of Compressed Concrete Elements Reinforced with FRP Bars

**DOI:** 10.3390/ma13194410

**Published:** 2020-10-03

**Authors:** Małgorzata Wydra, Maria Włodarczyk, Jadwiga Fangrat

**Affiliations:** 1 Faculty of Civil Engineering, Mechanics and Petrochemistry, Warsaw University of Technology, 09-400 Płock, Poland; 2 Faculty of Civil Engineering, Warsaw University of Technology, 00-611 Warsaw, Poland; maria.wlodarczyk@il.pw.edu.pl; 3 Building Research Institute, 00-611 Warsaw, Poland; j.fangrat@itb.pl

**Keywords:** basalt fiber reinforced polymer bars, GFRP bars, steel-reinforced columns, compressed concrete columns, nonlinear FEM

## Abstract

Although fiber reinforced polymer (FRP) bars have proved their usefulness in the case of reinforced concrete flexural elements, there are still limited data on their performance in such structures under compression. Despite multiple benefits of using FRP bars as the reinforcement in concrete elements, their potential application as main reinforcement in compressed elements is still very controversial, mainly due to the limited amount of published research results. The presented work partly fulfills this knowledge gap. Two series of theoretical analyses—one based on the stress distribution in the cross-section and the second using the finite elements method (FEM)—with reference to the experimental results are presented. The analyses concern basalt FRP, glass FRP, and steel-reinforced concrete elements under axial compression. There are derived calculations of load–displacement relations and stress values in bars. Damage progression was analyzed as well. Main findings are as follows: (1) a good agreement between calculated failure loads and experimental results has been achieved; (2) potential negative influence of FRP reinforcement on the compressive capacity of the reinforced element should not be neglected; (3) nonlinear FEM analysis is useful in predicting the maximum value of load and damage zones; (4) stress values of only about 100 MPa (much lower than their compressive strength value) were obtained in non-metallic bars. The results might be useful for the further establishment of design rules.

## 1. Introduction

Fiber reinforced polymer (FRP) composite bars are increasingly being used as an alternative to steel rebars in concrete structures. Their use is beneficial due to a higher corrosion resistance, tensile strength, electromagnetic indifference, and lower weight when compared to steel rods [1].

It is also worth mentioning that FRP bars are highly environmentally friendly material. Not only does the use of FRP materials lengthen the life cycle of a construction due to their high corrosion resistance [2,3,4], but also recycling processes are much easier when compared to elements with traditional reinforcement bars. Concerning steel reinforcement, the recycling process cannot be done unless the reinforcement is separated from the concrete. In the case of FRP reinforcement, a significant advantage is that concrete can be crushed with the bars inside; consequently, there is no need to remove them [5]. As an example, the environmental benefits to be gained from precast basalt fiber reinforced polymer (BFRP) reinforced concrete beams are highlighted in [6].

The most commonly used composite bars are made of:

- Glass fibers—glass fiber reinforced polymer (GFRP);
- Carbon fibers—carbon fiber reinforced polymer (CFRP);
- Aramid fibers—aramid fiber reinforced polymer (AFRP);
- Basalt fibers—basalt fiber reinforced polymer (BFRP);
- Hybrid (e.g., carbon/basalt) fibers—hybrid fiber reinforced polymer (HFRP).

The use of non-metallic bars in concrete elements increases the durability of the structure and reduces the amount of necessary repair work. Due to the high reliability of these types of bars, they are often used in components such as bridges and viaducts [7]. In addition, their high corrosion resistance makes them suitable for use in industrial constructions (e.g., electrolytic baths or transformer foundations) [4] and geotechnical facilities [8]. As the spectrum of applications of composite bars in concrete structures is constantly being expanded, intensive research is still needed. On the basis of analyses carried out so far, several national normative guidelines have been established:

- Japanese standard [9];
- Canadian standards for buildings [10] and bridges [11];
- American standard [12];
- Italian standard [13].

Both—the above standards and the research carried out so far—focus mainly on aspects related to bendable elements, such as slabs and beams (e.g., [4,14,15,16,17,18]). Analyses performed to date have proved that benefits from using FRP reinforcement must be balanced with taking into account their specific mechanical properties (such as lower elasticity modulus) in design procedures [1]. There is though only a limited amount of studies concerning compressed elements with internal FRP reinforcement. Some of the design codes even prohibit the use of FRP bars as the main reinforcement in compression. In many studies, the contribution of FRP bars to the Reinforced Concrete (RC) column capacity is consequently being neglected while there are also many studies suggesting the non-negative impact of using FRP bars in RC columns. The results of recent investigations in these opposite research directions have been summarized in [19].

In the article [19], there have been presented results from more than 300 tests published in 43 different experimental and analytical studies in the scientific literature. Three approaches of prediction ultimate compression strength of concrete columns have been derived and compared to above-mentioned experimental results: first one neglecting the contribution of FRP bars into the overall capacity of compressed concrete element, second: assuming percentage reduction in composite bars’ tensile strength in calculations and last one: basing on strength of FRP bars as a function of a certain level of concrete strain. Average ratio between predicted and experimentally obtained failure load was equal to 0.77, 0.97 and 0.94, respectively, for each method. 

However, in the above-mentioned review [19], it has also been pointed out that one of the future research needs in this area is further analysis of concrete columns with the use of internal BFRP bars, as their properties are not fully discovered yet. Although basalt composites are a relatively new material and extensive research on their properties is still needed [1,8,20,21], it has been already proved that using them is involved with multiple benefits when comparing to more common types of FRPs (such as GFRP or CFRP). Advantages of basalt fibers in regards to other types of fibers have been underlined in the article [22]. It is worth mentioning their advantages as follows: higher electrical insulation than in the case of glass fibers, high corrosion resistance and chemical stability, high acoustic and thermal insulation properties or good compatibility with metal, plastic and carbon fibers. The production of basalt fibers requires a lower amount of energy when comparing to glass fibers, which is a great benefit in terms of environmental impact [23].

This is the reason why BFRP composite bars have been chosen as one of the materials used in current research. Previous experimental studies [24,25] have shown that concrete elements reinforced with FRP significantly differ from the ones with metallic reinforcement in terms of their mechanical behavior. However, the potential for using composite bars as a replacement for steel bars has been proven, if proper design rules are established. A method of creating interaction curves (revealing safe combinations of normal load N and bending moment M) has been proposed in the article [25].

In this article, the influence of BFRP and GFRP reinforcement bars on the behavior of axially compressed concrete elements has been studied. The results have been compared with reference to steel-reinforced elements. Nonlinear finite element analysis has been provided with reference to the experimental results and analytical method basing on stresses balance in the cross-section.

## 2. Materials and Preparation of the Specimens

**Figure 1:**
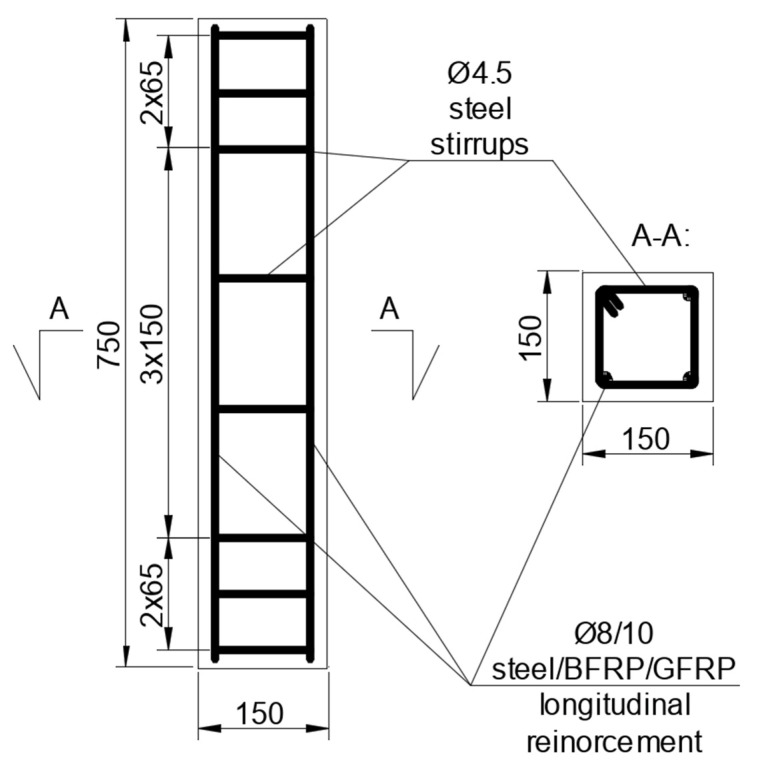
Reinforcement scheme, dimensions in mm.

Tests and analyses concern axially loaded concrete elements with different reinforcement types. The sets of the specimens have been summarized in Table 1. There was one specimen of each designation used. The designation of the additional specimen is also included, for which only analytical and FEM analysis has been carried out (no experimental data were available—specimen B10 ^1^). Dimensions of the elements are 150 mm × 150 mm × 750 mm. The reinforcement scheme is shown in Figure 1.

Concrete has been used with the medium value of compressive strength established experimentally on 150 mm concrete cubes as per [26] on *f_cm,cube_* = 41 MPa. It corresponds to the value *f_cm_* = 32.8 MPa, when concerning the maximum value of the concrete stress in the cross-section of the reinforced element [27]. Characteristic value of concrete compressive strength in uniaxial compression was assumed as *f_ck_* = 25 MPa. The cubic specimens have been stored in air-dried conditions before the compression tests—in the same way as the reinforced specimens in the experimental part.

Stirrups in all specimens were made of Rb500W steel [28]. Three types of longitudinal reinforcement materials have been used. Their mechanical parameters are described in Table 2.

## 3. Methods

### 3.1. Experimental Investigation

**Figure 2:**
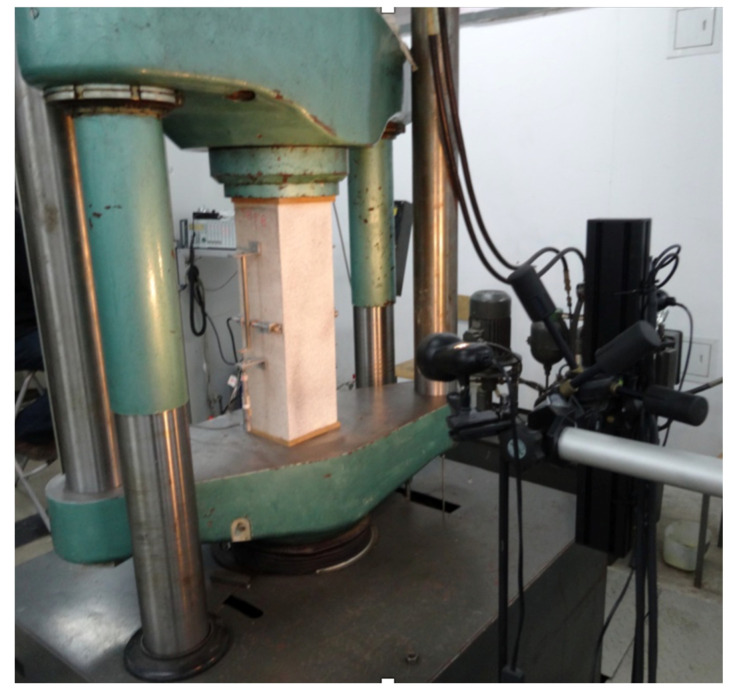
Test stand with the sample prior to the test.

The stand (Figure 2) consisted of the press (EU 1000), Aramis appliance (for digital image correlation analysis) and strain gauge measurement system (for the measurement of stresses in bars). Concrete elements were positioned axially in the machine. At each end, pads were placed (made of wood chipboard plates) in order to reduce stresses caused by direct pressure of steel plates on the concrete element. Specimens were tested under a static mechanical load till failure and destructive force values for each type of reinforcement have been identified. Once the critical point of the load was reached, the mechanical load was sustained in order to obtain a full course of the static equilibrium path, also in the postcritical stadium. The damage form has also been analyzed.

### 3.2. Analytical Method

For the purposes of the analytical method, the stress-strain relation for concrete derived by an Equation (1) has been used [27]:

(1) $$\sigma_{c}=\{\begin{array}{c} f_{cm}\left[1-{\left(1-\frac{\varepsilon_{c}}{\varepsilon_{c2}}\right)}^{n}\right]\;\mathrm{for}\;0\leq \varepsilon_{c}\leq \varepsilon_{c2}\\ f_{cm}\;\mathrm{for}\;\varepsilon_{c2}\leq \varepsilon_{c}\leq \varepsilon_{cu2} \end{array}$$

where:

- *f_cm_*—compressive strength of concrete (32.8 MPa);
- *n*—2 for concrete not higher class than C50/60;
- *ε_c_*_2_—minimum value of strain, for which the stresses gain compressive strength value, 2‰ for concrete not higher class than C50/60;
- *ε_cu_*_2_—maximum value of concrete strain, 3.5‰ for concrete not higher class than C50/60.

Constitutive model of steel was assumed as elastic-plastic with the parameters shown in Table 2. As the one of main difference between steel and FRP bars is that composites do not have yield strength, elastic model has been applied for them. Relation between strains and stresses in bars are linear in full range till failure in this case (modulus of elasticity assumed as per Table 2).

The maximum value of load has been calculated based on the balance of loads in the reinforced cross-section [26]. Preliminary analysis of eccentrically loaded FRP-reinforced columns is described in further detail by a co-author’s earlier contribution [25]. The equations used are presented below—Equations (2) and (3). The following designations have been used:

- *N*—normal force;
- *M*—bending moment;
- *A_si_*—area of singular bar;
- *σ_si_*—stress value in a singular bar;
- *n*—quantity of bars;
- *σ_c_*—stress value in concrete;
- *A_cc_*—compressed concrete area;
- *ν_2_*—the distance from the center of gravity of the concrete cross-section to the most compressed edge;
- *a_i_*—spacing of reinforcement from the most compressed section edge;
- *a_c_*—the location of the resultant force of the compressive stress block taken from the area *A_cc_*, measured from the compressed edge of the cross-section.

(2) $$N=\sum_{i=1}^{n}A_{si}\sigma_{si}+\iint_{A_{cc}}^{}\sigma_{c}dA_{cc},$$

(3) $$M=\sum_{i=1}^{n}A_{si}\sigma_{si}\left(\nu_{2}-a_{i}\right)+\iint_{A_{cc}}^{}\sigma_{c}dA_{cc}\left(\nu_{2}-a_{c}\right).$$

**Figure 3:**
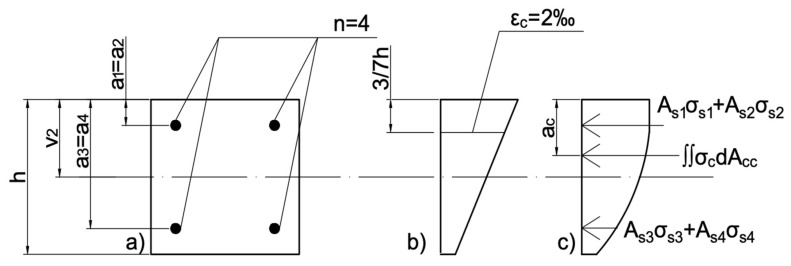
(**a**) Cross-section of the specimen. (**b**) Strain distribution in the cross-section. (**c**) Stress distribution in the cross-section.

Above described designations concerning strain and stress distribution are presented in Figure 3:

Assuming no eccentricity of the load (no bending moment), the strains in the cross-section with symmetrical reinforcement could be assumed to have the same value—*ε_c_*_2_ = 2‰—in the whole, equally compressed cross-section.

In the case of the metallic bars method, based on Equations (2) and (3), it is commonly used to assume maximum values of loads in the cross-sections for design purposes. In the case of non-metallic reinforcement, there is still not enough data confirming experimentally the influence of FRP reinforcement on the capacity of the element. In this article, two values of the capacity of the element have been proposed and analyzed:

- without considering the influence of composite bars (cross-section in the calculations consisting of concrete only—values given in brackets in Table 3);
- considering the influence of composite bars (assuming the value of stresses in the bars on the basis of their modulus of elasticity—as for metallic bars before gaining yield strength).

The values of strains in the ultimate limit state (for little values of load eccentricity) can be calculated as follows:

(4) $$\varepsilon =a\left(x-\frac{\varepsilon_{c2}}{\varepsilon_{cu2}}h\right)+\varepsilon_{c2}$$

and

(5) ε≤3.5‰,

where:

- *x*—distance from the point on the height of the cross-section to the most compressed section edge;
- *h*—height of the cross-section;
- *ε_c_*_2_ = 2‰;
- *ε_cu_*_2_ = 3.5‰;
- *a*—calibrating parameter.

The values of stresses have been calculated for various strain distributions (obtained by changing values of calibrating parameter *a*). The stress values in the bars have been calculated on the basis of their modulus of elasticity, while concrete relations described by Equation (2) have been used. Afterwards, values of *M* and *N* have been calculated as per Equations (2) and (3) and the value of eccentricity *e* has been calculated according to the following formula:

(6) $$e=\frac{M}{N}.$$

The results of *N* have been presented for the values of *e* equal to 1 cm and 2 cm.

### 3.3. Numerical Analysis

**Figure 4:**
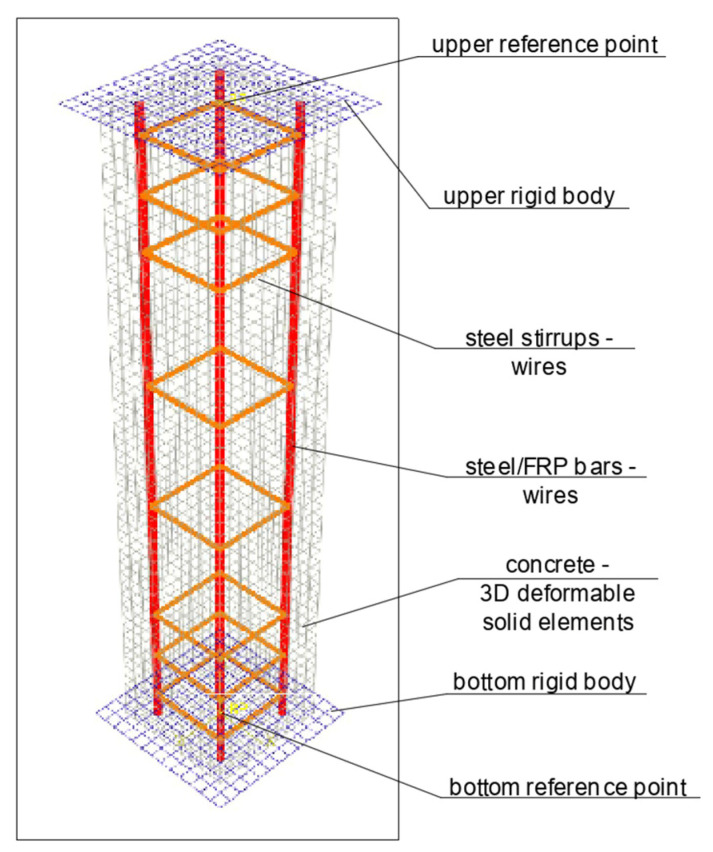
Model of the specimen created for the purpose of nonlinear analysis in the ABAQUS program.

In Figure 4, all types of parts used to create an assembly in the ABAQUS program are shown. Eight-node 3D solid deformable elements (type C3DR8) have been used for concrete, while two-node 3D deformable wire elements (type B31—beam) have been used for transverse and longitudinal reinforcement. Loading plates of the press have been modeled with the use of discrete rigid shell elements.

Steel and composite wires are modeled as embedded inside the concrete body. Interaction between rigid bodies and concrete elements has been assumed as hard contact in a normal direction and with frictional coefficient 0.2 in the tangential direction. The mesh size was 15 mm.

The following stress-strain relation for concrete in the compression range has been used [27]:

(7) $$\frac{\sigma_{c}}{f_{cm}}=\frac{k\eta -\eta^{2}}{1+\left(k-2\right)\eta }\;\mathrm{for}\;0<\varepsilon_{c}<\varepsilon_{c1},$$

where:

- $\eta =\frac{\varepsilon_{c}}{\varepsilon_{c1}}$;
- $\varepsilon_{c1}$—value of strain, for which the maximum value of stress occurs (2.1‰ for the concrete with the medium value of strength ~33 MPa);
- $\varepsilon_{cu1}$—maximum value of a concrete strain (3.5‰ for concrete not higher class than C50/60);
- $k=1.05\;E_{cm}\frac{\varepsilon_{c1}}{f_{cm}}$.

Linear growth of concrete stresses until failure has been assumed in the tensile range. Modulus of elasticity and tensile strength of concrete has been established by following equations [27]:

(8) $$E_{cm}=22\;{\left(0.1f_{cm}\right)}^{0.3}=\;31.4\;\mathrm{GPa},$$

(9) $$f_{ctm}=0.30\;{\left(f_{cm}-8\;\mathrm{MPa}\right)}^{\left(2/3\right)}=\;2.55\;\mathrm{MPa}.$$

Additionally, the Concrete Damaged Plasticity (CDP) model widely described in [29] was applied. CDP model is a modification of the Drucker–Prager plasticity hypothesis. The boundary surface of stresses is in that case described by the following parameters:

- Dilatation angle—assumed value in following FEM analysis: 36;
- Eccentricity—0.1;
- *f_b_*_0_/*f_c_*_0_—1.16;
- *κ*—0.667;
- Viscosity parameter—0.

Parameters for the damage evolution analysis (locating zones in the concrete part, in which degradation process propagates) have been calculated as per [30]. Damage parameters *d_c_* and *d_t_* are defined on the basis of the following equations:

(10) $$\sigma_{c}=\left(1-d_{c}\right)E_{0}\left(\varepsilon_{c}-\varepsilon_{c}^{pl}\right),$$

(11) $$\sigma_{c}=\left(1-d_{t}\right)E_{0}\left(\varepsilon_{t}-\varepsilon_{t}^{pl}\right),$$

where:

- *E*_0_—undamaged modulus of deformation;
- $\varepsilon_{c}$—compression strain;
- $\varepsilon_{t}$—tensile strain;
- $\varepsilon_{c}^{pl}$—plastic compression strain;
- $\varepsilon_{t}^{pl}$—plastic tensile strain.

Same assumptions as in the analytical model (see Section 3.2) have been adopted when concerning steel and FRP bars’ constitutive models.

Deformations of the specimens during the test were simulated by controlling displacements and rotations in reference points. Axial compression deformations were provided by moving down the upper reference point (and as a result pushing the upper rigid body onto the concrete element). Geometric nonlinearity has been considered. 

Apart from axially compressed element simulation, there has also been carried out analyses of eccentricity influence (load moved 1 cm and 2 cm from the center of the cross-section along the axis *x*).

After carrying out the simulations, they have been analyzed according to their:

- Values of reaction force as a function of displacement in upper reference point;
- Values of stresses in the middle of the longitudinal reinforcement bar as a function of reaction in upper reference point;
- Location of damaged zones through the test duration.

## 4. Results

### 4.1. Failure Load

In Table 3, the summarized results for the maximum values of load are presented:

- Achieved during the experimental investigation—*N_exp_*;
- Calculated with the use of analytical method *N_R_* (values in brackets assume no additional positive influence of the FRP bars on the overall capacity of the element);
- Obtained from FEM analysis in ABAQUS *N_FEM_*.

An analysis (Table 4) has been conducted into the influence of the imperfections by assuming additional eccentricity of a load equaling 1 and 2 cm.

Additionally, probabilistic analyses in usual designing procedures give additional safety reserves. It is related to the fact that values of material strength are intentionally lower than expected medium values. Therefore, in Table 4 authors show also values of loads calculated on the basis of characteristic values of materials’ mechanical parameters (*N**_Rc_***) instead of medium values used in all other calculations in this article.

The influence of an unintentional eccentricity of the load should be taken into consideration when designing steel-reinforced elements according to [27], and should not be less than 2 cm. For the above-presented results assume an even smaller eccentricity—1 cm—and most of the analytical results have lower values of failure loads than experimentally obtained ones, which provides some safety reserve for such calculations.

### 4.2. Load–Displacement Analysis

**Figure 5:**
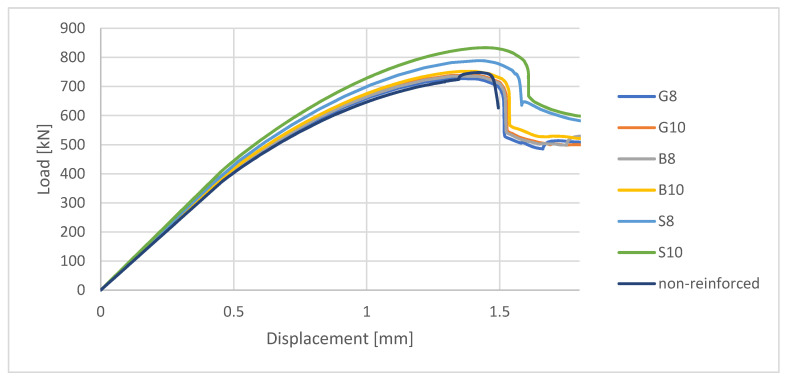
Load–displacement curve based on FEM analysis results.

In Figure 5, the relation between load and displacement of the upper rigid body during the simulation in ABAQUS for axially compressed specimens is shown. Before gaining approximately half the maximum value of load the course of the curve is almost linear, while it becomes then non-linear. Such non-linearity may correspond to the plasticity processes that can be observed in enlarging damaged areas of concrete. It has been described in further detail in Section 4.3.

After gaining the maximum value of the load, a sudden decrease in load value can be observed. The sudden drop in the capacity might be related to the fact that at that moment of FEM simulation a highly compressed area appeared in the middle height of the specimen. Plasticization processes of concrete propagated vividly and damage parameter in that area increased. This highly compressed area is easily visible on the damage map for post-failure stage (see Table 5). As a result, a part of the concrete cross-section was no longer available to sustain a load, therefore further deformation of the compressed element resulted in transferring stresses mainly by the bars.

The load–displacement curve for FRP-reinforced specimens reveals some similarities to the non-reinforced specimen in terms of curve course as far as the maximum value of the load. In some cases, even the negative impact of FRP bars on the capacity of the element might be observed. The main difference, though, concerns the post-failure state. When concerning the non-reinforced element, the analysis is interrupted just after gaining the maximum value of the load, while FRP-reinforced (as well as steel-reinforced) elements could still be able to sustain a reduced value of the load.

### 4.3. Damage Evolution and Failure Mode

**Figure 6:**
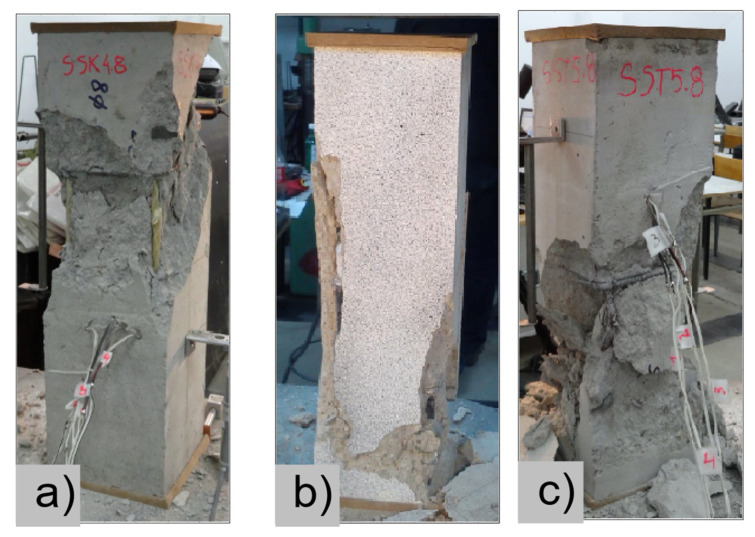
Typical forms of damage, specimens: (**a**) G8, (**b**) B8, (**c**) S8.

Figure 6 reveals failure modes observed in experimental investigations for specimens with the same diameter of the longitudinal bar and different types of reinforcement material. A brief description of failure progression in FRP-reinforced specimens is also provided below.

Concerning the G8 specimen (Figure 6a), the failure mode was sudden. A visible destruction started in the upper part of the specimen by detaching the concrete cover. While the maximum value of load was achieved, the crushing of concrete in the middle height part of the element occurred. In the B8 specimen (Figure 6b), initiation of failure was observed in the middle of the element. As soon as the maximum value of load was obtained, the concrete cover detached.

In Table 5, the summarized results of damage evolution simulations carried out in ABAQUS are shown. Maps of damaged zones represent three situations, when values higher than zero were firstly gained during the simulation (start of the damage process), when the element achieved a maximum value of the load and right after the failure (after rapid reduction in force value). The specimens are shown as a cross-sectional view. The first appearance of damage zones might correspond to the point on the load–displacement curve, when the course starts to reveal non-linearity (approximately half of the maximum load—see Figure 5). Damage zones in the following analysis are located mostly in two areas:

- Corners of the specimen (it may correspond to the detaching of the concrete cover observed in experimental investigations);
- Medium height of the specimen—most significant in the failure stadium (also revealed in the experimental test).

The maps of damage zones, with the failure of the BFRP-reinforced specimens (designation: B8), are shown in Table 6. In this case, the load eccentricity influence has also been considered. In the case of an eccentrically loaded specimen’s failure, propagation zones were much more intensive on one side of the specimen. Failure modes observed during tests (compare with Figure 6) are significantly more similar to the simulated for axially loaded specimens.

### 4.4. Stress Values in Longitudinal Reinforcement

**Figure 7:**
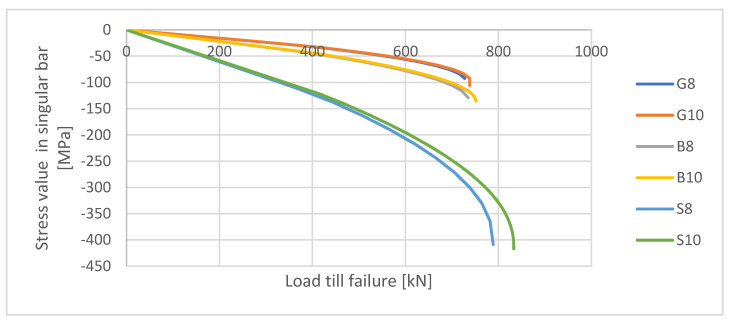
Stress values in singular bars were calculated in ABAQUS (FEM).

In Figure 7, the stress values in singular bars calculated in ABAQUS until the maximum value of the load are presented. It can be noticed that in the near failure stage dependence between stress in bar and load becomes non-linear. It can be a result of the plasticity and damage propagation processes occurring in concrete. The growth of stresses can be also observed and depends more on the type of the material (especially its modulus of elasticity) than on the diameter of the bar.

The maximum values of stresses in bars calculated in this simulation are much lower than the compressive strength of FRP. Therefore, one of the composite’s main beneficial characteristics (high strength) has a minor significance in the case of axially compressed reinforced concrete elements.

## 5. Discussion and Conclusions

FRPs have already been proven to be a material with multiple advantages (high corrosion resistance, tensile strength, electromagnetic indifference, lower weight, and environmental friendliness). The benefits of using composite bars as a replacement for steel reinforcement have been confirmed in multiple applications for the elements under flexural load. As there is still only a limited amount of studies concerning compressed elements with internal FRP reinforcement, intensive research is needed in this area in order to establish proper design procedures.

In this article, there are provided results of experimental and theoretical analyses of compressed concrete elements reinforced with BFRP, GFRP, and steel bars. In particular, their influence on the behavior of axially compressed concrete columns was examined.

Perfect axial compression is almost impossible in the real behavior of the construction element—the load is expected to be moved from the center of the cross-section due to various factors. One of these factors might be the imperfection of a specimen preparation. Typically, the influence of second-order effects should also be considered as created deformations can increase the eccentricity of the load effect. In this article, theoretically assumed concentric compression has been compared to the results obtained for the small values of load eccentricity—1 and 2 cm (Table 4). In this case, it has been assumed that the cross-sections after deformation remain flat.

Differences between calculated and measured failure loads are, in the case of the FEM model, at the level of up to 6% (Table 3) for most of the specimens (apart from specimen S10). In the case of the analytical method, differences are not higher than 9% (apart from specimen S10), which is also quite an accurate assessment. In both cases, the calculated load values are higher than those obtained in the experiment. Such differences might be influenced by the fact that models created for the purposes of calculations are always idealizations of reality, while in real tests multiple imperfections appear (such as dimension tolerances, material heterogeneity and eccentricity of load). 

Assumption of even small eccentricity (equaling 1 and 2 cm) resulted in reduction in calculated failure force values, therefore in most cases they were lower than obtained experimentally (apart from specimen S10—see Table 4). In above-mentioned analyses, medium values of mechanical parameters were used, while in the design procedures their values are initially lowered by using characteristic or design values. Assuming the characteristic value of materials’ mechanical parameters resulted in gaining analytical values of failure load lower than those obtained experimentally for all the specimens (even in case of specimen S10), which provides a safety reserve.

It has been established analytically that damage processes for both metallic and non-metallic reinforced elements started for load value of about half their compression capacity (see Table 5). This process resulted in nonlinearities of the load–displacement curve (Figure 5) and in relation between the stress in singular bar to external load (Figure 7). In the further deformation process, the damage zones continued to increase, therefore the non-linearity of above-mentioned curves can be seen in whole course till failure of the element.

Failure processes observed in the experimental investigation started by either concrete cover detaching at the top of the specimen or concrete crushing in the middle height of the concrete element (see Figure 6). Same damage progression areas can be noticed in the analysis carried out in ABAQUS (see Table 5). Moreover, the rapid degradation of load value revealed in Figure 5 can be related to extensive concrete crushing in the middle height of the specimen (compare to the Table 5: damage progression simulated in ABAQUS and Figure 6: failure modes in the experimental investigation). Damage parameter in that area increased vividly after failure due to intensification of plasticization processes. Further deformation of the compressed element resulted in decrease in load value as part of the concrete cross-section was no longer available to transfer loads. Afterwards, the stresses started to be transferred mostly by the bars.

Analytical investigation of FRP-reinforced axially compressed elements has proven that there is only a slight difference between reinforced and non-reinforced elements in terms of maximum value of load (see Table 3). Moreover, even the negative impact of composite reinforcement on the maximum load value in FEM analysis has been noticed in comparison to non-reinforced element (see Figure 5). However, both FRP-reinforced and steel-reinforced elements revealed some residual capacity after failure.

The main conclusions of performed analyses are as follows:

(1) A good agreement between calculated failure loads and experimental results has been proven for most of the analyzed specimens. The difference (up to 10%, apart from specimen S10) in the maximum value of load might be influenced by geometric tolerances (for example eccentricity of load, tolerances of dimensions or concrete cover thickness, etc.). When designing axially compressed elements, the influence of unintentional load eccentricity (not less than 2 cm [27]) should be taken into consideration. The above-presented analysis has proven that all the calculated values are higher than achieved in the experiment, only when eccentricity is assumed to be 0 cm. Considering eccentricity proves to give lower values of loads than established experimentally, even if the eccentricity equals only 1 cm, which seems to give a safety reserve.
(2) When comparing values of loads calculated with the use of FEM analysis for non-reinforced and FRP-reinforced elements, FRP compressed elements have in some cases lower values of load. The potential negative influence of FRP reinforcement on the compressive capacity of the reinforced element should not be neglected. A positive influence on the compressive capacity for other specimens has been calculated as not higher than 5%. On the basis of the load–displacement curve (Figure 5), it should be pointed out that post-critical stadium proves some residual capacity for FRP-reinforced elements (same for steel-reinforced ones), while non-reinforced elements crush rapidly after gaining a maximum load value.
(3) Nonlinear FEM analysis has appeared to be useful in predicting the maximum value of load as far as damage zones, in which plasticity processes and cracking might start and propagate for FRP- and metallic-reinforced compressed elements.
(4) Stress values of only about 100 MPa-much lower than FRPs’ compressive strength, have been obtained in bars for above-described analyses. Therefore, the benefits of using non-metallic reinforcement in compressed concrete elements should be seen in their high corrosive resistance, electrical indifference, or ecological friendliness rather than in their high strength. However, analyses of interaction between FRP bars and different types of concrete (with various values of elasticity modulus) are also recommended.

The analytical and FEM, as well as the experimental results, presented in this article have given valuable data on FRP compressed elements’ behavior. However, results for only six specimens have been considered. Additional experimental and analytical investigations should be carried out so the validity of the presented methods could be confirmed. After such validation, the proper designing rules could be established. Some analogies to currently existing design rules for metallic-reinforced compressed elements might be implemented, for example, concrete stress distributions or necessity of considering the unintentional eccentricity of a load.

## Figures and Tables

**Table 1:** Summary of analyzed reinforced specimens.

Reinforcement Material	Diameter of the Bars [mm]	Quantity of Reinforcement Bars	Designation of the Specimens	Reinforcement Ratio [%]
GFRP	8	4	G8	0.89
GFRP	10	4	G10	1.40
BFRP	8	4	B8	0.89
BFRP	10	4	B10 ^1^	1.40
steel	8	4	S8	0.89
steel	10	4	S10	1.40

^1^ No experimental data were available.

**Table 2:** Mechanical characteristics of longitudinal reinforcement materials [2,28].

Reinforcement Material	Poisson’s Ratio	Modulus of Elasticity *E* [GPa]	Yield Strength *f_y_* [MPa]	Ultimate Tensile Strength *f_ut_* [MPa]
GFRP	0.25	50	-	up to 1600
BFRP	0.25	70	-	1100
steel	0.3	200	500	550

**Table 3:** Results of experimental investigations, analytical method, and finite elements method (FEM) analysis.

Reinforcement Type	Designation of the Specimen	Experimental Value *N_exp_* [kN]	Analytical Failure Load *N_R_* [kN]	*N_exp_/N_R_*	FEM Failure Load *N_FEM_* [kN]	*N_exp_/N_FEM_*
4#8 GFRP	G8	699.1	753.2 (742.9) ^2^	0.928 (0.941) ^2^	727.9	0.960
4#10 GFRP	G10	733.2	764.5 (742.9) ^2^	0.959 (0.987) ^2^	738.3	0.993
4#8 BFRP	B8	727.2	761.2 (742.9) ^2^	0.955 (0.979)^2^	735.2	0.989
4#10 BFRP	B10^1^	-^1^	777.1 (742.9) ^2^	-^1^	752.2	-^1^
4#8 steel	S8	740.0	813.5	0.910	789.0	0.938
4#10 steel	S10	580.0	858.7	0.657	833.1	0.696

^1^ No experimental data were available. ^2^ The values in brackets are calculated with the assumption of no influence of fiber reinforced polymer (FRP) bars on the overall compression capacity.

**Table 4:** The influence of load eccentricity and characteristic values of materials’ parameters on the calculated failure load.

Designation of the Specimen	Load Eccentricity *e*									
	*e* = 0 cm				*e* = 1 cm			*e* = 2 cm		
	*N_exp_* [kN]	*N_R_* [kN]	*N_Rc_* [kN]	*N_FEM_* [kN]	*N_R_* [kN]	*N_Rc_* [kN]	*N_FEM_* [kN]	*N_R_* [kN]	*N_Rc_* [kN]	*N_FEM_* [kN]
G8	699.1	753.2 (742.9) ^2^	578.9 (566.3) ^2^	727.9	654.8	503.9	629.2	576.1	443.9	543.5
G10	733.2	764.5 (742.9) ^2^	590.2 (566.3) ^2^	738.3	666.1	515.2	630.3	587.4	455.2	538.4
B8	727.2	761.2 (742.9) ^2^	586.9 (566.3)^2^	735.2	662.8	511.9	630.0	584.1	451.9	540.4
B10 ^1^	-^1^	777.1 (742.9) ^2^	602.07 (566.3) ^2^	752.2	678.7	527.7	631.5	599.9	460.2	558.0
S8	740.0	813.5	639.2	789.0	705.3	556.7	705.0	616.7	489.2	607.1
S10	580.0	858.7	684.4	833.1	750.5	594.4	745.0	652.1	519.4	645.0

^1^ No experimental data were available. ^2^ The values in brackets are calculated with the assumption of no influence of FRP bars on the overall compression capacity.

**Table 5:** Damage evolution simulated in ABAQUS for axially compressed elements (specimens G8 and S8).

Part of the Testing Process	G8 Specimen (GFRP Bars)		S8 Specimen (Steel Bars)	
	Compression Damage Zones	Tension Damage Zones	Compression Damage Zones	Tension Damage Zones
First occurrence of visible damage zones	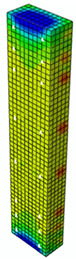 Load value F = 422.4 kN	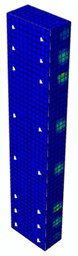 Load value F = 422.4 kN	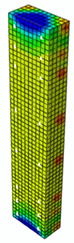 Load value F = 442.0 kN	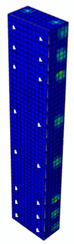 Load value F = 442.0 kN
Failure	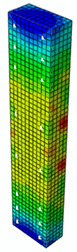 Load value F = 727.9 kN	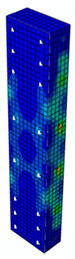 Load value F = 727.9 kN	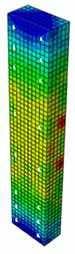 Load value F = 789.0 kN	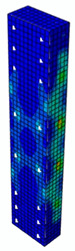 Load value F = 789.0 kN
Post - failure	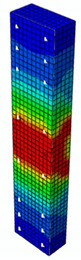 Load value F = 500.0 kN	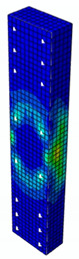 Load value F = 500.0 kN	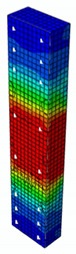 Load value F = 550.8 kN	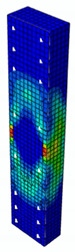 Load value F = 550.8 kN

**Table 6:** Maps of damage zones for failure stage simulated in ABAQUS (specimen B8)—analysis of the load eccentricity influence.

Load Eccentricity	*e* = 0 cm (F = 735.2 kN)	*e* = 1 cm (F = 630.0 kN)	*e* = 2 cm (F = 540.4 kN)
Compression damage zones	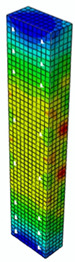	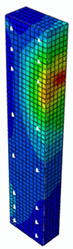	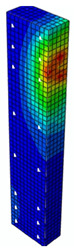
Tension damage zones